# Camurati-Engelmann Disease: Unique Variant Featuring a Novel Mutation in *TGFβ1* Encoding Transforming Growth Factor Beta 1 and a Missense Change in *TNFSF11* Encoding RANK Ligand

**DOI:** 10.1002/jbmr.283

**Published:** 2010-11-04

**Authors:** Michael P Whyte, William G Totty, Deborah V Novack, Xiafang Zhang, Deborah Wenkert, Steven Mumm

**Affiliations:** 1 Center for Metabolic Bone Disease and Molecular Research, Shriners Hospital for ChildrenSt. Louis, MO, USA; 2 Division of Bone and Mineral Diseases, Washington University School of Medicine at Barnes-Jewish HospitalSt. Louis, MO, USA; 3 Section of Musculoskeletal Radiology, Mallinckrodt Institute of Radiology, Washington University School of MedicineSt. Louis, MO, USA; 4 Department of Pathology, Washington University School of Medicine at Barnes-Jewish HospitalSt. Louis, MO, USA

**Keywords:** DEAFNESS, DIAPHYSEAL DYSPLASIA, OSTEOMALACIA, OSTEOSCLEROSIS, PERIOSTITIS, PREDNISONE, TORUS PALATINUS

## Abstract

We report a 32-year-old man and his 59-year-old mother with a unique and extensive variant of Camurati-Engelmann disease (CED) featuring histopathological changes of osteomalacia and alterations within *TGFβ1* and *TNFSF11* encoding TGFβ1 and RANKL, respectively. He suffered leg pain and weakness since childhood and reportedly grew until his late 20s, reaching 7 feet in height. He had deafness, perforated nasal septum, torus palatinus, disproportionately long limbs with knock-knees, low muscle mass, and pseudoclubbing. Radiographs revealed generalized skeletal abnormalities, including wide bones and cortical and trabecular bone thickening in keeping with CED, except that long bone ends were also affected. Lumbar spine and hip BMD Z-scores were + 7.7 and + 4.4, respectively. Biochemical markers of bone turnover were elevated. Hypocalciuria accompanied low serum 25-hydroxyvitamin D (25[OH]D) levels. Pituitary hypogonadism and low serum insulin-like growth factor (IGF)-1 were present. Karyotype was normal. Despite vitamin D repletion, iliac crest histology revealed severe osteomalacia. Exon 1 of *TNFRSF11A* (RANK), exons 2, 3, and 4 of *LRP5*, and all coding exons and adjacent mRNA splice junctions of *TNFRSF11B* (OPG), *SQSTM1* (sequestosome 1), and *TNSALP* (tissue nonspecific alkaline phosphatase) were intact. His asymptomatic and less dysmorphic 5′11″ mother, also with low serum 25(OH)D, had milder clinical, radiological, biochemical, and histopathological findings. Both individuals were heterozygous for a novel 12-bp duplication (c.27_38dup, p.L10_L13dup) in exon 1 of *TGFβ1,* predicting four additional leucine residues in the latency-associated-peptide segment of TGFβ1, consistent with CED. The son was also homozygous for a single base transversion in *TNFSF11*, predicting a nonconservative amino acid change (c.107C > G, p.Pro36Arg) in the intracellular domain of RANKL that was heterozygous in his nonconsanguineous parents. This *TNFSF11* variant was not found in the SNP Database, nor in published *TNFSF11* association studies, but it occurred in four of the 134 *TNFSF11* alleles (3.0%) we tested randomly among individuals without CED. Perhaps the unique phenotype of this CED family is conditioned by altered RANKL activity. © 2011 American Society for Bone and Mineral Research.

## Introduction

Camurati-Engelmann disease (CED: OMIM #131300) was characterized by Cockayne in 1920.(1) In 1922, Camurati suggested that the disorder was heritable.(2) Seven years later, Engelmann described the severe form.(3) In 1948, Neuhauser et al.(4) coined “progressive diaphyseal dysplasia” to emphasize the advancing diaphyseal hyperostosis, yet normal metaphyses and epiphyses, seen radiographically.

Descriptions of approximately 200 CED patients(5,6) show that both genders equally and all races are affected.(6) However, the penetrance (including age of onset, rate of progression, and clinical course) is inexplicably variable.(7,8) Usually, CED manifests during childhood with leg pain, limping or a broad-based and waddling gait, muscle wasting, fatigue, weakness, and decreased subcutaneous fat in the limbs that can mimic a muscular dystrophy.(6) Severe CED features a characteristic marfanoid(9) body habitus, occasionally including tall stature,(10,11) enlarged head with a prominent forehead and proptosis, and thin and disproportionately long limbs(6) with palpably widened bones and skeletal tenderness. Cranial nerve palsies(12) or raised intracranial pressure can also develop.(13,14) Sometimes puberty is delayed,(13,15) and there may be secondary hypogonadism(16) and prolonged growth.(6) Deafness is not uncommon.(6,12) Hips and knees can develop flexion contractures.(9,15) Some patients have Raynaud's phenomenon, hepatosplenomegaly, and other findings suggestive of vasculitis.(17) Remission of symptoms has been reported during adult life.(13,18–20)

The principal radiographic finding in CED is hyperostosis of major long bone diaphyses due to proliferation of osseous tissue on periosteal and endosteal surfaces.(4) Maturation of the new bone accentuates the cortical thickening. The tibiae and femora are most commonly involved; less frequently are the radii, ulnae, humeri, and short tubular bones. Hyperostosis usually begins in the diaphysis, is fairly symmetrical, and spreads slowly, sometimes to include the metaphyses.(11,21–24) However, epiphyses are characteristically spared.(10,13,20,21,23,25) Typically, long bone shafts gradually widen and develop irregular surfaces. Osteosclerosis can also involve the skull and axial skeleton, including clavicles, scapulae, and pelvis. In severely affected children, nearly the entire skeleton is altered, and some areas appear osteopenic.(15,26,27) Bone scanning usually reveals increased radionuclide uptake within active lesions,(28) again sparing the epiphyses.(29) Substantial radioisotope accumulation with minimal radiographic findings characterizes early and active disease,(30,31) yet some “carriers” manifest only bone scanning abnormalities.(32) Magnetic resonance imaging (MRI) shows no marrow disturbance.(33)

Electron microscopy of CED muscle may reveal myopathic and vascular changes, including atrophy of type II fibers without degenerative changes.(5) Skeletal histopathology of CED features new bone formation along diaphyses where nascent, disorganized osseous tissue undergoes centripetal maturation and incorporation into the cortex.(34) Osteomalacia has not been reported.(20)

Routine biochemical parameters of mineral homeostasis are typically normal in CED, although markers of bone turnover,(35) including serum alkaline phosphatase (ALP), can be elevated.(19,34) When present, modest hypocalcemia and significant hypocalciuria seem to reflect a markedly positive calcium balance.(19) Mild anemia, leukopenia, and elevated erythrocyte sedimentation rate (ESR) are consistent with a systemic disturbance.(17) CED can respond to glucocorticoids,(6,36–39) showing diminution of bone pain and correction of histological abnormalities in bone when small doses of prednisone are given on alternate days.(36,38,40) The clinical and laboratory findings in severe CED, and the disorder's responsiveness to glucocorticoid therapy, indicate an inflammatory connective tissue disease.(17) Dihydrotachysterol treatment has also been beneficial.(24) Various bisphosphonates may help,(41) but more likely will not,(14,26) and they are known to have exacerbated CED bone pain.(27,42)

In 2000, the cause of CED was discovered to be mutation within one region (the latency-associated-peptide portion) of the gene that encodes transforming growth factor β1 (*TGFβ1*).(43,44) These mutations in *TGFβ1* seem to enhance TGFβ1 anabolic effects within bone by freeing TGFβ1 from the latency-associated-peptide (see Discussion).(6) To date, 10 distinctive mutations in this domain have been identified.(6,30) Nevertheless, there is evidence for locus heterogeneity causing CED.(45)

We report a mother and adult son with a unique variant of CED featuring a generalized skeletal disorder with the histopathological characteristics of osteomalacia and alterations in two genes that importantly regulate skeletal homeostasis: *TGFβ1* and *TNFSF11*, encoding TGFβ1 and RANK ligand (RANKL), respectively.

## Materials and Methods

### Family report

The propositus and each of his parents gave written informed consent to be studied, as approved by the Human Research Protection Office, Washington University School of Medicine, St. Louis, MO.

#### Patient 1 (son)

This exceptionally tall 32-year-old white man, an only child, was self-referred in 2007 to diagnose his skeletal disease. He was accompanied by his nonconsanguineous parents.

At birth, he was full-term, delivered vaginally, and weighed 7 pounds, 13 ounces (50th percentile) and was 21.5 inches long (90th percentile).

He was well until approximately age 10 years, when his mother noted that his ankles became pronated and flat feet “appeared overnight.” His limbs then grew disproportionately long, and he experienced difficulty running and keeping up in gym class. Both legs ached at night. Several physicians had said that radiographs showed osteopathia striata.

He reported that he grew until at least age 24 years, reaching 7 feet tall, and that an endocrinologist had excluded acromegaly. His physes were said to be open in his late 20s, but growth charts and radiographs from that time were not available. Knock-knees with flexion contractures caused an arduous and shuffling gait. He could walk barefoot for 10 minutes before his knees and ankles hurt. Pain persisted in his feet despite shoe orthotics. After prolonged sitting, his ankles would swell “over his shoes.” Mobic (meloxicam), taken until one year earlier, improved his mobility, generalized soreness, flexibility, and periodic leg, shoulder, and back pains. Ibuprofen was almost as effective.

His mother reported that he had had finger clubbing since childhood, but there did not seem to be a cardiopulmonary explanation, in that he had a normal echocardiogram and no pulmonary symptoms.

At age 26 years, deafness was discovered, and hearing aids helped thereafter.

His gait, thin body build, and clubbed fingertips had not changed during the past five years. Constant “sinus congestion” was worse in the morning.

**Fig. 1:**
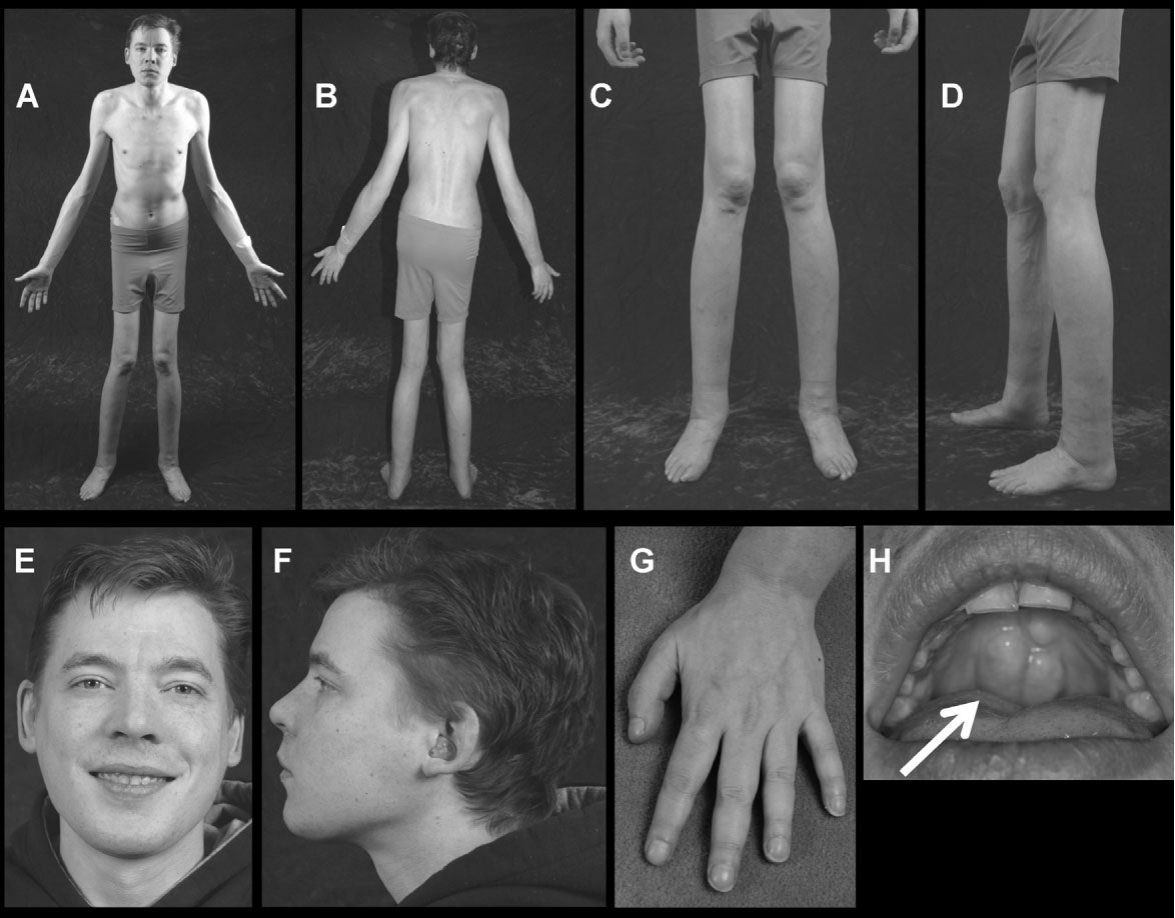
Patient 1 (son) (*A,B*) This 32-year-old, youthful-appearing, hypogonadal man stands 6′10″ tall and has disproportionately long arms and legs. However, his upper segment is of normal size; the extremities are excessively long. (*C,D*) He has knock-knee deformity, relatively small feet, little muscle mass, and wide bones. (*E,F*) Despite generalized and severe skeletal disease, his face is not dysmorphic. Hearing aids are worn bilaterally for deafness since age 26 years. (*G*) Pseudoclubbing has been apparent since childhood. (*H*) A torus palatinus (exostosis) is present (arrow).

Physical examination revealed an exceptionally tall and thin man with knock-knees and hip and knee contractions that caused stooped posture (Fig. 1). Nevertheless, his height was 6 feet, 10 inches (Z-score + 4.5) measured using a Harpenden stadiometer (Holtain, Crymmych, UK). Arm span measured 6 feet, 9 inches (reduced by slight elbow contractures), sitting height 3 feet, 1 inch (Z-score 0), and head circumference 59 cm (∼ 92%). Limbs were disproportionately long with little muscle mass. Weight was 187 lb (body mass index [BMI] 18.6 kg/m^2^). His face was not dysmorphic, but facial hair was minimal (Fig. 1). His jaw was not broad or prognathic, yet there was a torus palatinus. The teeth were carious, and there was gingivitis. A prominent perforated nasal septum (no history of trauma or cocaine use) involved the inferior cartilaginous septum. With hearing aids, he perceived tuning fork vibration better on the left. No tenderness accompanied fist percussion over his spine or compression of his ribs. His chest was not deformed. Forearms were broad from expanded bones but nontender. His fingers had wider bones than joints and considerable clubbing. His feet were remarkably small for his extreme height (shoe size 11) (Fig. 1).

Otolaryngology consultation revealed narrow external auditory canals, severe ossicular fixation, sensorineural hearing loss, and minimal internal auditory canal narrowing with mild nerve compression secondary to bone disease. A sleep study showed severe periodic limb movement disorder accompanying his history consistent with restless leg syndrome.

#### Patient 2 (mother)

This 59-year-old woman was discovered, following a car accident in college, to have “strange bones.” A skeletal survey reportedly showed “benign osteopathia striata,” but the radiographs could not be located for review.

In high school she was thin and not athletic, with excessively long lower limbs and little muscle mass. Menarche was at age 13 years, yet she reportedly grew until age 17 years, with irregular periods until starting birth control pills. Menopause was at age 55 years.

She considered herself well and had not fractured. Some increasing stiffness occurred with aging, primarily in her back, hips, and lower limbs, including her knees. She, too, wore arch supports. However, symptoms of arthritis occurred only in her ankles, where swelling sometimes followed prolonged sitting. Severe neck pain for eight months benefited from physical therapy. L_4_-L_5_ disc surgery helped back pain following a fall. She recounted no finger clubbing, but there was expansion of the proximal interphalangeal joints of her right hand. Her dentition was good. She claimed only minimal hearing loss since age 50 years, but audiometry was > 10 years previously. She wore eyeglasses until successful Lasik surgery at age 40 years.

The subject had taken sulfasalazine for ulcerative colitis since age 30 years, as well as macrodantin because of leukocytes in her urine. She had not received glucocorticoids but took folic acid, other vitamins, and fish oil for the colitis and sulfasalazine treatment. She had taken a calcium supplement sporadically for five years.

**Fig. 2:**
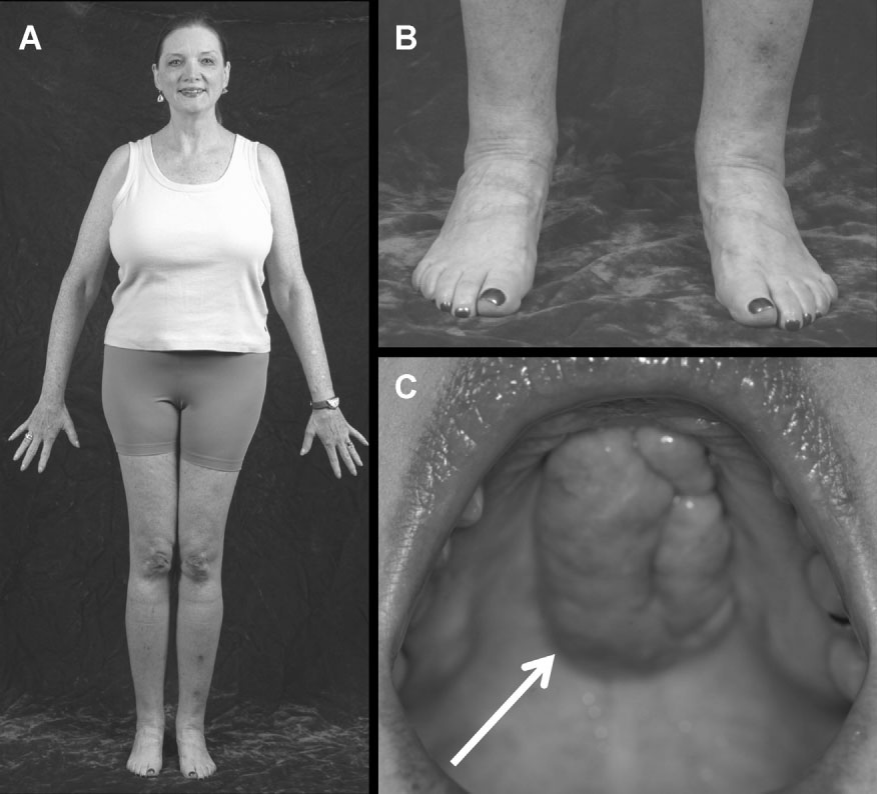
Patient 2 (mother) (*A*) This 59-year-old woman is 5′11″ tall and has disproportionately long lower extremities, but less strikingly so than her son. (*B*) Some muscle wasting is present, with wide bones at the ankles. (*C*) A torus palatinus (exostosis) is present (arrow).

Physical examination revealed height of 5 feet, 10½ inches (Z-score + 2.5) (Fig. 2). Her healthy sister was said to be 5 feet, 7 inches tall. Arm span was 5 feet, 11½ inches (Z-score + 2.8), sitting height 2 feet, 11 inches (Z-score 0), head circumference 58 cm (∼ 90%), and weight 181 pounds. Torus palatinus was present (Fig. 2). Teeth appeared well formed. She could hear a softly ticking watch. No tenderness accompanied fist percussion of her spine or femora, compression of her ribs, or gentle squeezing of her forearms. Some tenderness occurred with percussion of the pretibial regions. The distal tibias were expanded with little overlying soft tissue. Feet were not flat. Fingers showed some clubbing. Crepitus (2–3+) occurred on passive flexion and extension of her knees, but not with rotation of the ankles.

#### Father

This 59-year-old man had no history of bone or joint complaints. Height was 5 feet, 11 inches (Z-score + 0.4), arm span 6 feet, 2 inches (Z-score + 1.4). His sitting height and head circumference were identical to his son's.

### Biochemical studies

Routine biochemical studies were undertaken at the Washington University Medical Center, St. Louis, MO, and LabCorp (Kansas City, MO, USA). Urine N-telopeptide (NTX) was measured at Mayo Medical Laboratories, Rochester, MN, USA. At Shriners Hospital for Children, St. Louis, MO, USA, assays included serum 25-hydroxyvitamin D (25[OH]D; Immunodiagnostic Systems, Tyne and Wear, UK), bone-specific ALP (BAP; Quidel Corp., San Diego, CA, USA), osteocalcin (kit LKON1, Siemens Healthcare Diagnostics, Los Angeles, CA, USA), and tartrate-resistant acid phosphatase (TRAP-5b; kit 8033, Quidel, Los Angeles, CA, USA). In 2007, 15 healthy adults provided fasting serum to calculate reference values (normal = Nl) for these assays.(46)

### Radiological studies

We attempted to obtain all previous radiologic studies of our patients for review. Mother and son underwent a limited radiographic skeletal survey. DXA was performed using a Hologic Discovery C (S/N 70311) instrument (Hologic, Waltham, MA, USA).

### Iliac crest histology

Serum 25(OH)D was low in both son and mother, at 12 and 13 ng/mL in the months of January and October, respectively (30–100 Nl) (see below). The son, therefore, took one 50,000-unit capsule of vitamin D_2_ (ergocalciferol) weekly for 6 weeks before transiliac crest biopsy. The mother was not supplemented. Both received two 3-day courses of tetracycline hydrochloride (250 q.i.d.), separated by approximately 2 weeks and ending 3 days before biopsy using a Bordier 5 mm internal diameter trephine.

Bone sections were stained with Goldner trichrome to identify osteoid and osteoblasts, with von Kossa to distinguish calcified from noncalcified bone (for better assessment for osteoid), and unstained sections were used for fluorescence microscopy to search for tetracycline labels.

### Gene mutation studies

Genomic DNA was purified from blood leukocytes using the Gentra Puregene DNA extraction kit (Invitrogen, Carlsbad, CA, USA).

Exon 1 of *TNFRSF11A*, where activating duplications cause familial expansile osteolysis and other allelic disorders that resemble Paget bone disease,(47) was amplified by PCR and sequenced according to published methods.(48) Exons 2, 3, and 4 of *LRP5*, where activating mutations cause a high-bone-mass phenotype often associated with torus palatinus, were also sequenced according to published methods.(49) Furthermore, all coding exons and adjacent mRNA splice sites of *TNFRSF11B* (osteoprotegerin; OPG), *TNFSF11* (RANKL), *SQSTM1* (sequestosome 1), *TNSALP* (tissue nonspecific alkaline phosphatase), and *TGFβ1* were amplified by PCR and sequenced in both directions using published methods and primers(50,51) and unpublished primers (available on request) to search for mutations that cause juvenile Paget disease, osteopetrosis, Paget bone disease, hypophosphatasia, and CED.

DNA sequences were evaluated using AlignX software (Vector NTI, Invitrogen, Carlsbad, CA, USA), and by inspecting individual electropherograms.

## Results

### Biochemical findings

Biochemical testing of mineral and skeletal homeostasis indicated accelerated bone turnover in both patients, but especially in the son (Table 1).

**Table 1:** Biochemical Findings

	Serum										Urine	
		
	Calcium	Phos	ALB	Crt	PTH	ALP	BAP	25(OH)D	OCN	TRAP	Calcium	NTX
Patient 1 (Son)	9.3	4.2	3.8	0.5	36	170	87	31	232	4.3	< 36	1225
NORMAL RANGE	8.6–10.3 mg/dL	2.3–4.3 mg/dL	3.6–5.0 g/dL	0.7–1.5 mg/dL	14–72 pg/mL	38–126 U/L	15–41 U/L	32–100 ng/mL	9.4–47.4 pg/mL	2.7–5.4 U/L	50–250 mg/24 hr	21– 66 nmol/ mmol Crt
Patient 2 (Mother)	10.0	3.5	4.5	0.7	28	143	ND	13	63	ND	142	93
NORMAL RANGE	8.5–10.6 mg/dL	2.5–4.5 mg/dL	3.5– 5.5 g/dL	0.5–1.5 mg/dL	12–65 pg/mL	25–150 U/L		32–100 ng/mL	3.1–13.7 pg/mL		50–250 mg/24 hr	19–63 nmol/ mmol Crt

ND = Not done.

#### Patient 1 (son)

Although serum 25(OH)D was initially low at 12 ng/mL (30–100 Nl), total and ionized calcium, magnesium, PTH, and albumin were normal (Table 1). Hypocalciuria was noted at < 39 mg/gm creatinine (50–250 Nl). Serum ALP was elevated at 170 U/L (38–126 Nl). His subnormal serum creatinine of 0.5 mg/dL (0.7–1.5 Nl) perhaps reflected low muscle mass. Plasma protein was slightly elevated at 8.7 g/dL (6.5–8.5 Nl). Serum total bilirubin was low at 0.2 mg/dL (0.3–1.1 Nl). Serum testosterone was deficient at 152 ng/dL (241–827 Nl) while follicle stimulating hormone was 1.6 IU/L (1.4–18 Nl for men), prolactin 5.2 ng/mL (2.1–18.0 Nl), and 17β-estradiol < 40 pg/mL (< 52 Nl for men). Serum random growth hormone was elevated at 2.17 ng/mL (0.01–0.97 Nl), but IGF-1 was low at 69 ng/mL (115–307 Nl) (Mayo Clinic Department of Laboratory Medicine and Pathology), suggesting undernutrition. However, serum ferritin was 66 ng/mL (22–322 Nl).

Erythrocyte sedimentation rate (ESR) was 67 mm/hr (0–12 Nl) and serum C-reactive protein (CRP; high sensitivity) was 57 mg/L (> 10 mg/L consistent with infection or inflammation). Antinuclear antibody was negative.

Markers of bone turnover, including serum ALP, indicated rapid skeletal remodeling (Table 1). Serum osteocalcin was 232 pg/mL (3.1–13.7 Nl) and BAP was 87 U/L (15.0–41.3 Nl). Urine NTX was 1225 nmol bone collagen equivalents (BCE)/mmol creatinine (21–66 Nl). Significant hypocalciuria (see above) may have reflected positive calcium balance from his skeletal disease compounded by vitamin D deficiency. Although serum 25(OH)D was initially low, it was 31 ng/mL (30–100 Nl) after ergocalciferol supplementation at the time of iliac crest biopsy. Still, his urine calcium remained undetectable.

#### Patient 2 (mother)

Although serum 25(OH)D was low at 13 ng/mL (32–100 Nl), total and ionized calcium, inorganic phosphate (Pi), PTH, and ALP were normal (Table 1). A 24-hr urine collection contained 142 mg calcium (50–250 Nl); i.e., 115 mg calcium/gm creatinine in keeping with sequential DXA studies that indicated stable BMD (see below).

Serum osteocalcin was elevated at 63 ng/mL (9–47 Nl), including a subsequent measurement at Shriners Hospital of 45 ng/mL (3–14 Nl). Urine NTX was high at 99 nmol BCE/mmol creatinine (19–63 Nl).

Serum creatinine, prolactin, T_4_, and TSH were normal (Lab Corp, Kansas City, MO). Her ESR was 23 mm/hr (0–35 Nl) and CRP was 1.9 mg/L (0–3 Nl).

### Radiological findings

Findings are reported per patient and anatomic region.

#### Patient 1 (son)

**Fig. 3:**
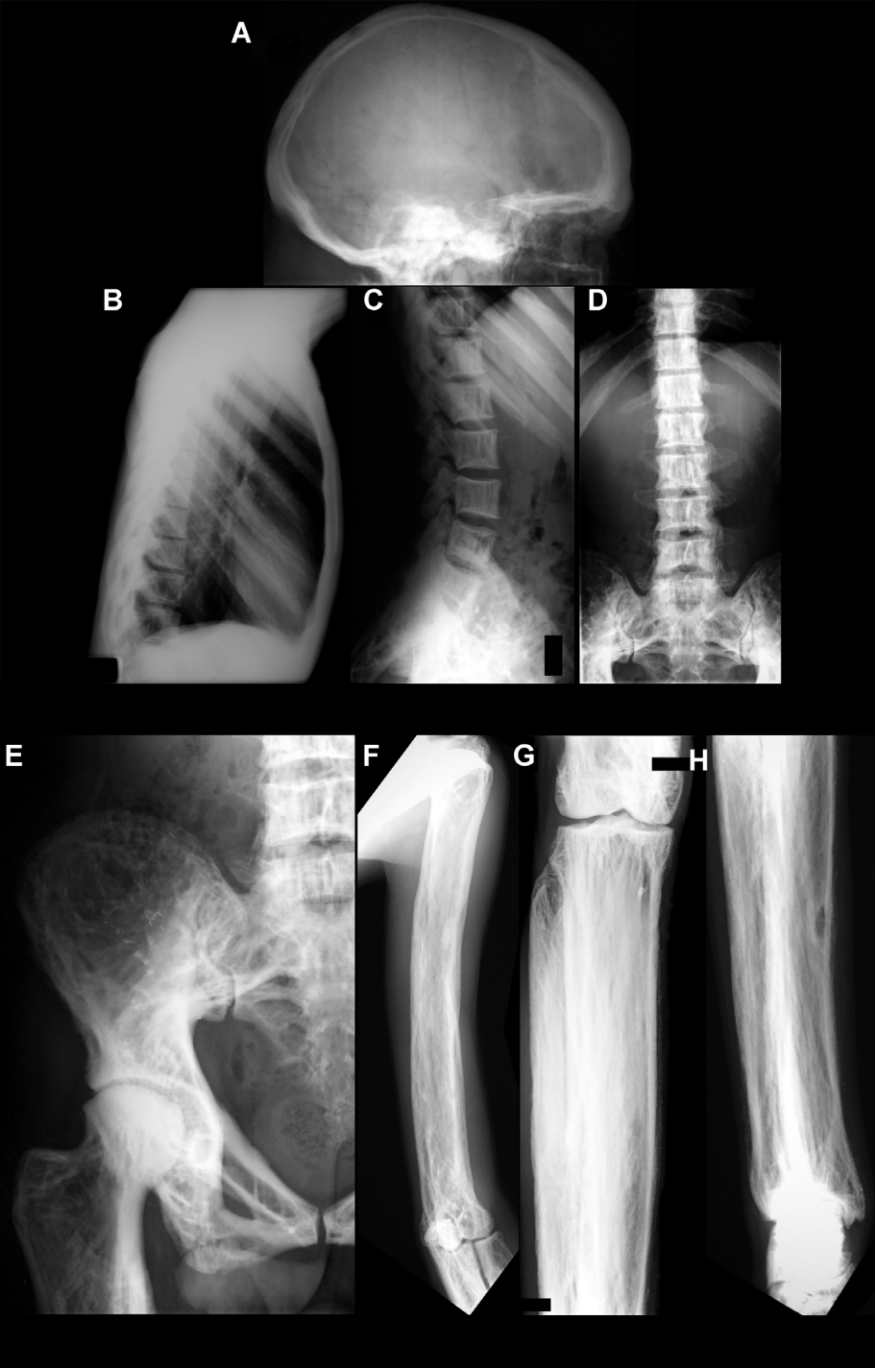
Radiographic findings of Patient 1 (son): Diffuse osteoslcerosis is present throughout the skeleton. (*A*) Lateral radiograph of the skull at age 32 years shows frontal calvarial thickening and sclerosis. The parietal, temporal, and occipital bones are largely spared. Normal intramedullary space is lost throughout the calvarium. (*B*) Lateral radiograph of the chest shows cortical and trabecular thickening throughout uniformly expanded ribs. Vertebral bodies are not enlarged but show similar thickening of the cortices and trabeculae and are diffusely radiodense. (*C,D*) Thickening of the cortex affects both the vertebral body and its posterior elements, but with minimal enlargement of the bodies and only moderate expansion of the posterior elements. A shared change affects the medullary space of the vertebral bodies and their posterior elements, producing coarse, thick, vertically oriented trabeculae in a pattern reminiscent of vertebral hemangiomas. (*E*) Anteroposterior radiograph of the right hemipelvis shows changes similar to those in the spine, with diffuse, mild bone expansion and cortical and trabecular thickening that follows normal stress line patterns, and are not as disorganized as seen in Paget bone disease. Marked cortical thickening and uniform sclerosis is particularly prominent in the superior and inferior pubic ramus, with no cortical splitting or “tram-tracking.” Cartilage loss and spurring in both hips indicates moderate osteoarthritis. (*F-H*) Anteroposterior radiographs of the upper (*F*) and lower extremities (*G,H*) show similar changes throughout the long tubular bones. There is diffuse bone expansion with cortical changes including cortical thickening, periosteitis that is more mature proximally than distally, and coarsening of the medullary trabecular pattern most prominent in the metaphyses and epiphyses. Bone shape is almost normal at the epiphyses, where there is no expansion or deformity. No significant arthritic change was noted in the shoulders, elbows, wrists, knees, or ankles. Notably, the periostitis extends to the ends of the long tubular bones in a pattern similar to primary hypertrophic osteoarthropathy (pachydermoperiostosis). Bone expansion also involves the short tubular bones of the hands with changes in the trabecular pattern resembling the long tubular bones (not shown).

Skeletal survey in 2007 showed abnormalities nearly throughout the skeleton (Fig. 3).

The lateral skull had frontal calvarial thickening and sclerosis producing frontal bossing. The parietal, temporal, and occipital bones were largely spared, with only minimally increased radiodensity, but the normal intramedullary space was absent throughout the calvarium. Limited visualization of the facial bones showed no sclerosis or deformity.

Anteroposterior and lateral radiographs of the cervical, thoracic, and lumbar spine showed a common pattern of abnormality involving the entirety of every vertebral segment. The vertebral bodies were diffusely radiodense, with thickening of the cortex of both the vertebral body proper and the posterior elements. However, there was minimal enlargement of the vertebral bodies themselves and only moderate expansion of the posterior elements. A common pattern of marked trabecular thickening was present within the medullary space of the vertebral bodies and the posterior elements, producing coarse, thick, vertically oriented trabeculae in a pattern reminiscent of vertebral hemangiomas. The cortical and trabecular changes were not associated with significant scoliosis or kyphosis, and the intervertebral disk spaces in the cervical and thoracic spine remained normal. Disk narrowing indicative of disk degeneration was noted at L_4_-L_5_ and L_5_-S_1_. No evidence of marginal spurring or facet arthritis was present. There was no indication of spondyloarthritis.

A chest radiograph from 2001 also revealed diffuse osteosclerosis. There was no indication of active cardiopulmonary disease. Cortical and trabecular thickening was present throughout the ribs, which were uniformly expanded. The vertebral bodies were not enlarged, but they showed similar thickening of the cortices and trabeculae. The findings in 2001 were unchanged in 2007.

Anteroposterior radiographs of the scapulae and pelvis, including the sacrum, showed changes similar to the spine with mild, diffuse bone expansion and cortical and trabecular thickening. The thickened trabeculae followed normal stress line patterns and were not as disorganized as typically seen in Paget bone disease. Marked cortical thickening and uniform sclerosis was particularly prominent in the superior and inferior pubic rami, without the cortical splitting or “tram-tracking” of Paget bone disease. Cartilage loss and spurring were present in both hips, indicating moderate hip osteoarthritis.

Anteroposterior radiographs of the upper and lower extremities showed similar changes throughout the long tubular bones. There was diffuse bone expansion with cortical changes, including thickening, periostitis more mature proximally than distally, and coarsening of the medullary trabecular pattern most prominent in the metaphyses and epiphyses. No significant arthritic change was noted in the shoulders, elbows, wrists, knees, or ankles. The bone shape was almost normal at the epiphyses, where there was no expansion or deformity. Bone expansion involved the short tubular bones of the hands, with similar changes in the trabecular pattern, as seen in the long tubular bones. Of particular note, the periostitis extended to the ends of the long tubular bones in a pattern similar to primary hypertrophic osteoarthropathy (pachydermoperiostosis), which can be an autosomal dominant disorder affecting males more severely than females.(52) Autosomal recessive pachydermoperiostosis is the result of disturbances in prostaglandin metabolism due to deactivating mutations in the gene that encodes 15-hydroxyprostaglandin dehydrogenase.(53)

DXA at age 32 years showed BMD Z-scores of + 7.7 and + 4.4 in the patient's L_1_-L_4_ spine and total hip, respectively. Although these measurements can be influenced by large body size and bone shaping, his sitting height and vertebral size were normal, and therefore his vertebrae did seem to be genuinely dense.

In 2008, thin-section CT of the temporal bones was performed bilaterally in the axial plane. Coronal sections of the paranasal sinuses were also obtained. Diffuse thickening and sclerosis was observed of all the visualized osseous structures of the calvaria and facial bones. The temporal bones were diffusely thickened and sclerotic, with a dysmorphic appearance. Underpneumatization affected the mastoid air cells — those seen were partially fluid filled, likely representing chronic mastoiditis. Minimal internal auditory canal narrowing occurred bilaterally. Inner ear ossicles were normal. Narrowing and decreased space within the middle ear cavity was due to dense osseous sclerosis. The middle ear cavity was diminutive, given the adjacent sclerotic and thickened temporal bone. The facial canals, cochlea, and semicircular canals appeared normal. Dural walls were thickened and sclerotic, but not to the severity of other calvarial bones. The superior orbital fissures were patent bilaterally. The frontal, ethmoid, sphenoid, and maxillary sinuses were clear.

#### Patient 2 (mother)

**Fig. 4:**
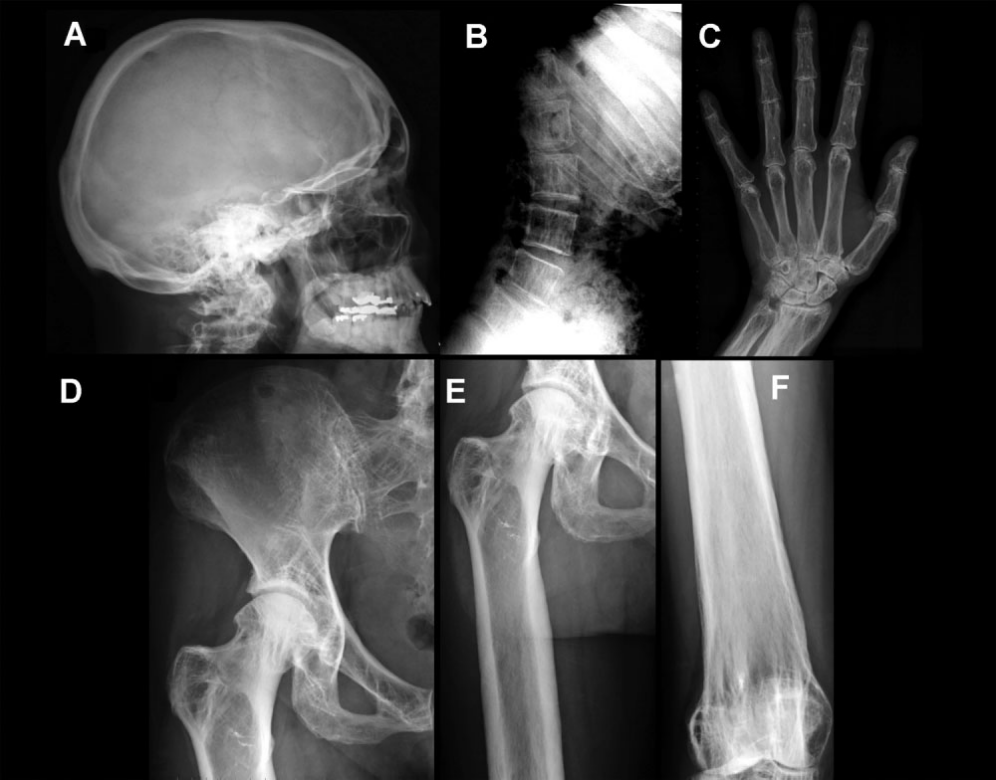
Radiographic findings of Patient 2 (mother) (*A-F*) The radiographic skeletal findings are in all ways similar to those of the son but are generally less severe. The skull is normal, with no osteosclerosis and a normal medullary space. The overall pattern of osteosclerosis and bone expansion is present, although there is less prominent vertebral body change and better tubulation of the long bones and less prominent trabecular thickening. There is less immature periostitis noted along the long tubular bones, although the mature periosteal reaction is again seen to extend to the epiphysis. As in the son, there are changes of moderate bilateral hip osteoarthritis and early lumbar degenerative disk disease.

Radiographic skeletal survey of the mother in 2007 showed findings that were in all ways similar to the son's but generally less severe (Fig. 4). The overall pattern of osteosclerosis and bone expansion was present, although with better tubulation of the long bones, less prominent trabecular thickening, and less significant vertebral body change. The skull was unremarkable, with no osteosclerosis and a normal medullary space. The short tubular bones of the feet were involved, but there was less immature periostitis over the long tubular bones, although the mature periosteal reaction was again seen to extend to the epiphysis. Like the son, the mother had changes of moderate, bilateral hip osteoarthritis and early lumbar degenerative disk disease. There was mild sacroiliac joint osteoarthritis but no indication of spondyloarthritis. MRI of the lumbar spine from 2004 did not clarify the bone abnormality but documented normal marrow, with no indication of a marrow infiltrative disease.

DXA at age 60 years revealed BMD Z-scores of + 2.6, + 4.4, and –0.2 in her L_1_-L_4_ spine, total hip, and wrist, respectively. Elsewhere, DXA (GE Lunar Corporation, Madison, WI, USA) had showed little change in L_1_-L_4_ spine and femoral neck BMD between ages 52 and 57 years.

### Histopathology findings

#### Patient 1 (son)

**Fig. 5:**
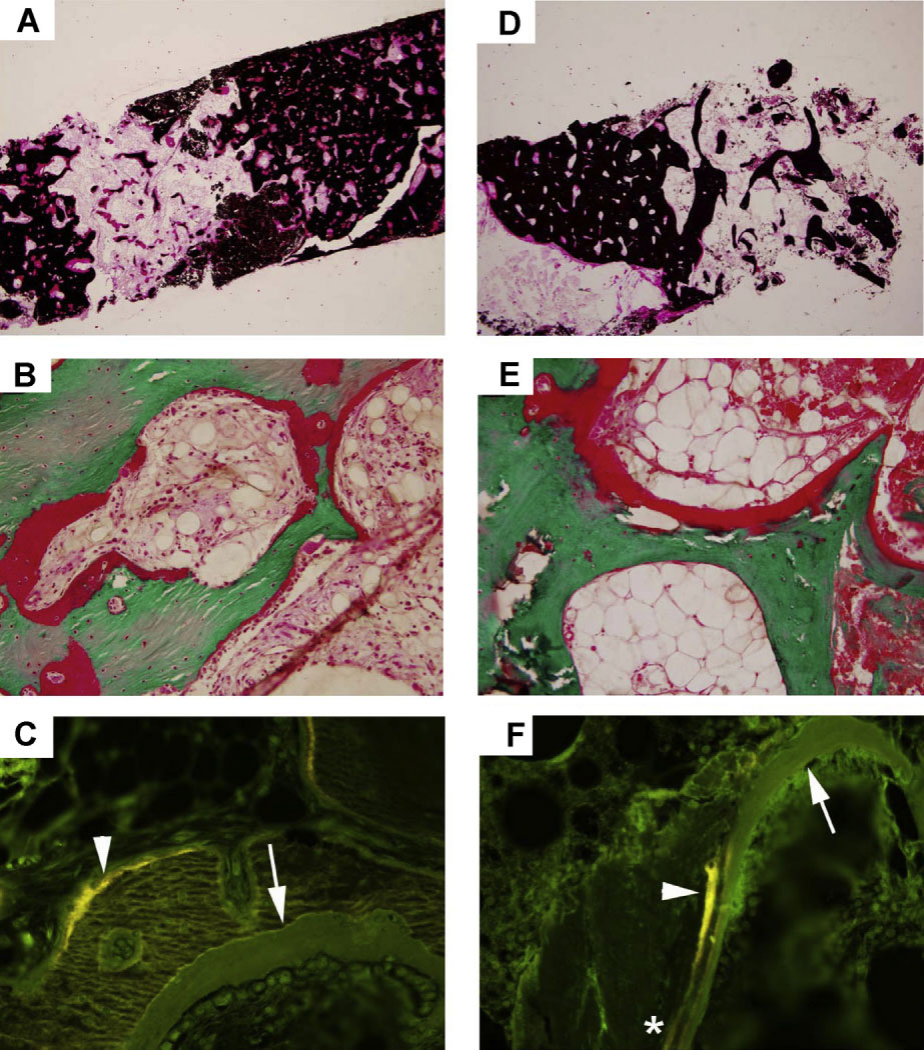
Histological findings: (*A-C*) The iliac crest specimens from patient 1 (son) and (*D–F*) patient 2 (mother). Overall, the findings were consistent with osteomalacia that was more striking in the son. (*A,D*) Von Kossa–stained sections show thickened porous cortices and decreased trabecular connectivity. (*B,E*) Goldner trichrome–stained sections show thick osteoid seams covering most trabecular and cortical surfaces. (*C,F*) Fluorescence microscopy shows broad single tetracycline labels (arrowheads) as well as unlabeled osteoid (arrows). Although double tetracycline labels could be found in both patients, they were more numerous in the mother (asterisk).

Von Kossa–stained sections showed two thickened cortices with very high porosity (Fig. 5*A*). Trabecular bone had very poor connectivity. There was a paucity of bone marrow elements. Both von Kossa and Goldner trichrome–stained sections showed extensive osteoid (Fig. 5*A, B*). In many places, the osteoid was covered by plump, active-appearing osteoblasts. Osteoclasts were also increased in some areas, most notably those without osteoid, but had normal appearances. There was no osteitis fibrosa. Fluorescence microscopy of an unstained section showed broad, wide, predominantly single labels, while many areas of osteoid showed no tetracycline labeling (Fig. 5*C*). Taken together, the findings indicated severe osteomalacia.

#### Patient 2 (mother)

Transiliac crest biopsy with von Kossa–stained sections showed a porous, thickened cortex and poor trabecular connectivity in the mother (Fig. 5*D*), although not to the degree seen in the son. Increased osteoid was also observed on von Kossa and Goldner trichrome–stained sections (Fig. 5*D, E*), which again was somewhat less extensive than in the son. Fluorescence microscopy of unstained sections showed more double labels than those of the son, but also areas of broad single labels and unlabeled osteoid indicating osteomalacia (Fig 5*F*).

### Mutation analyses

No mutations were detected in the son's genes encoding RANK, OPG, LRP5, sequestosome 1, or TNSALP. However, in *TGFβ1*, we found a novel 12-bp duplication (c.27_38dup, p.L10_L13dup) in exon 1 that would insert four leucine residues into the latency-associated peptide. This mutation was also detected in the mother. The duplication was reminiscent of a report of a 9-bp insertion at the same site, adding three leucine residues in a patient with CED.(44)

Additionally, we identified in the son's *TNFSF11* gene, encoding RANKL, a homozygous, single-base alteration predicting a nonconservative amino acid change (c.107C > G, p.Pro36Arg) in RANKL that was heterozygous in both parents. This *TNFSF11* transversion was not found in dbSNP (SNP Database),(54) nor was it reported in several association studies of RANKL polymorphisms.(55–57) However, we found it in 4 of 134 *TNFSF11* alleles (3.0%) tested randomly among our patients and family members who did not have skeletal disorders resembling CED.(58)

### Treatment

Patient 1's symptoms did not change with ergocalciferol supplementation and increases in serum 25(OH)D to > 30 ng/mL. However, there was significant improvement with a tapering course of prednisone that commenced with 40 mg orally daily (Howard Rosen, MD, personal communication). After several days, back pain resolved, after which he no longer used crutches at home and stopped analgesic medications. An alternate-day maintenance regimen of prednisone is a current goal for his treatment.

## Discussion

We report a unique disorder affecting a mother and her adult son that is a variant of CED where essentially the entire skeleton (including the ends of major long bones) is abnormal and histopathological changes are consistent with osteomalacia. The son's condition is distinctly worse considering his clinical, radiological, biochemical, and histopathological findings. A novel heterozygous *TGFβ1* duplication in keeping with CED was identified in both individuals, but the son is also homozygous and his mother heterozygous for a missense change in *TNFSF11* encoding RANKL.

### CED phenotype

The son manifests many of the typical features of CED reviewed in the Introduction, and his digital clubbing,(59) marfanoid body habitus,(9) flat feet, valgus ankles, and knock-knees sometimes also occur in this disorder.(15) Deafness, too, is prevalent with skull base involvement and may reflect a sensorineural, conductive, or mixed pathogenesis.(60,61) There can be narrowing of (1) the internal auditory canals and bony encroachment on nerves and vessels,(60) (2) the tympanic cavities with fixation or adhesion of ossicles, and (3) the bony part of the auditory tubes, causing serous otitis media.(60) Skeletal maturation can be delayed.(15) In 1964 Clauson and Loop(15) reported a “tall” boy whose growth plates were open and who was still growing at age 20 years. We have not, however, found reports of perforation of the nasal septum in CED.

The radiographic findings of CED were detailed by Neuhauser et al. in 1948.(4) Hyperostosis progresses from the diaphysis over the long axis of tubular bones in both directions.(4) Metaphyses become widened, and the distal femur may develop an Erlenmeyer flask deformity.(11) Epiphyses are often broadened, with flattening of the condylar surfaces at the knee. There is also general widening of the articular surfaces of the hips and elbows.(4) CT shows that, along diaphyses, abnormalities are not uniformly distributed.(11) Endosteal alterations are more pronounced than periosteal changes, and sclerosis is not homogeneous.(11) Although long bone ends are typically spared by the hyperostosis of CED, metaphyses and epiphyses may appear osteopenic.(15) Reports of epiphyseal involvement by osteosclerosis are exceptional, but have included the femoral capital epiphysis bilaterally and a proximal tibial epiphysis in a 73-year-old man.(62) Some authors conclude that epiphyses, indeed the entire skeleton, can eventually become involved.(6,15)

The radiographic pattern of our patients' skeletal disorder is not specific. Overall, it most closely resembles CED, with symmetrical, generalized osteosclerosis and tubular bone expansion primarily involving the metadiaphysis. However, the findings include more prominent involvement of the flat bones and spine, as well as more pronounced immature periostitis extending to the ends of the tubular bones. Additionally, in the son there is less well-developed medullary narrowing, more prominent trabecular thickening, and more cranial vault involvement than is typically seen in CED. The mother's changes are less striking.

Histological study of CED bone has shown thickened periosteum and small blood vessel walls without inflammation. The surface of lesional bone can consist of immature osseous tissue(63) where woven bone then undergoes maturation and cancellous compaction.(34) There can be dense, subjacent cortical bone with normal haversian systems and minimal osteoblastic and osteoclastic activity.(15,63,64) Osteoclasts may be few.(40) Marrow space can contain increased interstitial fibrosis.(15) Osteoid osteoma or chronic osteomyelitis have been diagnosed in CED.(65,66) In 2007, Bondestam et al.(27) reported iliac crest histology (following tetracycline labeling) of a 10-year-old boy with CED that featured trabecular osteoporosis with normal bone remodeling. Osteoporosis has been described by other patients with CED,(6) but we have not found a report of osteomalacia.

The principal histopathological abnormality of our patients' iliac crest specimens was not anticipated. Changes consistent with osteomalacia were documented in both individuals despite elevated biochemical markers of bone turnover indicating accelerated skeletal remodeling. The osteoid excess in the specimens did not appear to be due to rapid bone turnover. We saw primarily broad, single fluorescent labels as well as unlabeled osteoid and verified that both subjects took the tetracycline hydrochloride correctly. Vitamin D deficiency did not seem to explain their osteomalacia.(67) Mother and son each had a serum 25(OH)D level that might have been considered normal until recently.(67) Furthermore, their serum levels of calcium, Pi, and PTH were unremarkable. Additionally, the son took 300,000 units of vitamin D_2_ orally over the six weeks leading up to his biopsy, when he had a serum 25(OH)D level > 30 ng/mL. Serum ALP activity and other markers of bone turnover were elevated, especially in the son, perhaps originating not only from increased skeletal mass, but also from regions elsewhere of enhanced bone remodeling within lesional bone not captured by iliac crest biopsy. In the son's iliac crest specimen, pockets of normal-appearing osteoclasts were seen on bone surfaces not covered by osteoid, likely contributing to his especially elevated urine NTX level. His hypocalciuria, before and after vitamin D_2_ supplementation, likely reflected positive bone balance, not gastrointestinal malabsorption, although we do not have sequential BMD measurements by DXA to verify this.

### CED genetic defect

CED features variable penetrance and wide-ranging expressivity,(6) even within families.(6,7,66,68) Our patients' disorder was distinctly worse in the son. In 1997, Saraiva reported that the severity of CED seems to depend on gender and parental transmission, with males carrying a paternal mutation being the most affected.(66) In 2000, he(69) suggested that genetic “anticipation” (disease severity increasing over generations) might explain such variation. Perhaps gene repeat expansion, enhanced somehow by father-to-son transmission, was the mechanism.(69) That same year, Makita et al.(7) reported significant phenotypic variation within a three-generation Japanese family with CED.

In 2000, discovery that *TGFβ1* mutation causes CED, together with subsequent family studies, confirmed the disorder's variable expressivity by documenting asymptomatic family members.(43,44) Following reports of higher plasma TGFβ1 levels in individuals with a -509T allele, Campos-Xavier et al.(68) in 2001 investigated whether *TGFβ1* polymorphisms explain the variable penetrance of CED. A seemingly unaffected 18-year-old girl in the youngest generation of a Portuguese family(68) carried their R218H mutation in *TGFβ1*. These investigators(70) reported significant intrafamilial variability for CED, including five individuals within four families who did not have clinical manifestations despite a *TGFβ1* mutation. No significant association with polymorphisms was established among CED families.(68) In fact, two subjects with both the -509T polymorphism and a *TGFβ1* mutation showed either no clinical symptoms or severe disease. In 2004, Wallace et al.(6) also did not find effects from *TGFβ1* polymorphisms. Perhaps CED variability reflected modifying genes.(6,68) In 2006, Janssens et al.(30) studied 24 CED families and noted that their types of *TGFβ1* mutation would probably not cause anticipation. Instead, they suggested that additional genetic factors, such as single nucleotide polymorphisms (SNPs) in *TGFβ1* or alterations in other genes, might modulate CED expressivity.(30)

In fact, there may be CED variants without defects in *TGFβ1*. In 2002, Nishimura et al.(9) proposed a second form of CED (CED type II) featuring striations of the bones. For two unrelated 7- and 11-year-old Japanese girls with many clinical features of CED, radiographs showed cortical thickening resembling CED but also metaphyseal expansion of long bones; coarse and thick trabeculae of the long and short tubular bones; striations in the spine, pelvis, and long bones; and cranial sclerosis restricted to the petromastoid region. These changes overall were considered identical to the extremely rare disorder “hyperostosis generalisata with striations of the bones”(70) rather than to CED.(9) PCR direct sequencing of all exons and their flanking regions of *TGFβ1* did not reveal mutations in these girls. Furthermore, PCR single strand conformational polymorphism analysis of the TGF-β type 1 receptor gene (*TGFβR1)* did not show any aberrant DNA fragments. These authors concluded that their patients represented a unique entity, CED type II.(9)

Unlike in CED II, our patients have a distinctive *TGFβ1* mutation. Nevertheless, our cumulative findings support a potential impact of modifying genes. Perhaps their RANKL variant influences the CED phenotype and manifests an allele dosage effect (see below).

### TGFβ1 function

*TGFβ1* features a unique expression structure encoding a colinear protein from seven exons that must be cleaved into two peptides: a latency-associated protein (LAP) encoded by exons 1–5, and TGFβ1 encoded by exons 6–7.(71) Pre-pro-TGFβ1 consists of a signal peptide (1aa–27aa), the LAP region (33aa–252aa), and TGFβ1 (293aa–390aa).(71) The pro-TGFβ1 precursor molecule dimerizes and undergoes proteolytic cleavage to separate the LAP and TGFβ1 moieties. However, the LAP remains associated in the bone matrix with mature TGFβ1 until subjected to specific activation conditions.(30) Release of LAP activates circulating TGFβ1(30) and presumably also TGFβ1 within bone. Then, free TGFβ1 regulates bone cells(6) in multiple ways.

TGFβ1 stimulates skeletal turnover by increasing both bone formation and resorption.(6) In fact, biochemical markers of skeletal remodeling were elevated in our patients. TGFβ1 enhances the generation and activity of osteoblasts.(6) Osteoclasts are affected by two pathways. TGFβ1 directly promotes their formation and activity, but also indirectly inhibits osteoclastogenesis by increasing OPG and decreasing RANKL expression in osteoblasts and stromal cell precursors.(72,73)

Saito et al.(74) reported increased proliferation of human osteoblast MG-63 cells during coculture with CED fibroblasts, suggesting mutant (R218H) TGFβ1–stimulated osteoblast growth. McGowan et al.(75) used CED peripheral blood mononuclear cells with a R218C mutation and noted 5-fold osteoclast formation and 10-fold bone resorption that were inhibited by soluble TGFβ1 type II receptor. Therefore, these two missense mutations (near the cysteine residues involved in LAP dimerization) enhance TGFβ1 effects on both osteoblasts and osteoclasts, increasing bone turnover.(6) Accordingly, CED mutations are gain-of-function defects for the skeleton.(75) TGFβ1 can inhibit some myogenic transcription factors and adipogenesis,(76,77) perhaps explaining the weakness and cachectic phenotype of severe CED.(27)

### Our patients' TGFβ1 defect

All 10 *TGFβ1* mutations causing CED involve LAP; none are within TGFβ1 itself.(6) They comprise a limited set of defects: (1) a three-leucine insertion in the signal peptide (L10-L12dup), (2) a Y81H missense mutation encoded in exon 1, (3) a R156C missense mutation in exon 2, and (4) a group of at least seven missense mutations (R218C, R218H, H222D, C223R, C223S, C223G, C225R) in exon 4 at or near two cysteine residues involved in disulfide bonding between the LAP homodimers.(6,30) Because this last group of mutations tends to cluster at the C-terminus of LAP near the site of interchain disulfide bonds coupling the LAP homodimers,(6) perhaps they disrupt LAP binding to TGFβ1, leading to increased release of active TGFβ1 from cells.(6) Therefore, these heterozygous mutations seem to augment wild type, functional TGFβ1.

Our two patients have a heterozygous 12-bp duplication in exon 1 of *TGFβ1*, predicting a four-leucine duplication (L10-L13dup) at the same site previously reported for the three-leucine duplication.(44) Cell culture studies showed that the L10-L12dup mutation, which is in the signal peptide, seemed to compromise secretion, causing intracellular accumulation of TGFβ1.(71) This led to increased TGFβ1 activity by a different mechanism and potentially an alternative pathway, compared to the missense mutations near the cysteine residues involved in dimerization.(71) The leucine duplication of our patients would presumably function similarly in this second way.

### Other genes

Patient 1 had periosteal new bone formation that extended to the ends of his long tubular bones. This finding resembled pachydermoperiostitis (OMIM %167100, #259100), where clubbing and disproportionately long limbs can be a feature. However, he had no characteristic skin changes or acro-osteolysis, and we did not examine his 15-hydroxyprostaglandin dehydrogenase gene; this gene encodes the principal enzyme involved in prostaglandin degradation and is deactivated in autosomal recessive pachydermoperiostitis (#259100).(53)

Ghosal hematodiaphyseal dysplasia (OMIM #231095), an autosomal recessive disorder due to loss-of-function mutations in the *TBXAS1* gene encoding thromboxane synthase,(78) causes refractory anemia and skeletal dysplasia affecting diaphyses and metaphyses even more extensively than in CED.(79) Thromboxane synthase produces thromboxane A2 and thereby modulates expression of RANKL (increases) and OPG (decreases).(78) We did not measure serum RANKL or OPG levels in our patients.

### Our patients' RANKL variant

In addition to a distinctive *TGFβ1* mutation causing a four-leucine duplication, patient 1 (the son) also carries a homozygous missense variation (P36R) in RANKL. This *TNFSF11* change was not found in dbSNP(54) and was not reported in several association studies of RANKL SNPs with bone phenotypes.(55–57) The base change predicts a nonconservative amino acid alteration in a conserved region of this molecule that regulates osteoclastogenesis. RANKL is membrane bound, or soluble. P36 is part of an intracellular proline-rich region of RANKL (7 proline residues per 10–amino acid stretch) near the plasma membrane. The unique ring structure of proline gives it a special role in polypeptide conformation, and some proline-rich regions form important functional motifs; e.g., the 10–amino acid proline-rich motifs in Src homology 3 (SH3) binding sites(80) and a proline-rich region of p53 required for apoptosis.(81) However, we are unaware of any distinctive function for this 10–amino acid proline-rich region in RANKL. Alternatively, this transversion may inhibit RANKL trafficking to the cell membrane, insertion there, or shedding (Dr. Christopher Nelson, personal communication). We identified a single copy of this RANKL variant in four of 67 patients or their family members (134 alleles) with bone diseases other than CED. Preliminary studies of a healthy population detected the heterozygous change in 3 out of 103 individuals (3/206 alleles) (Mumm and Villareal, unpublished). Accordingly, this missense change may represent a relatively rare RANKL polymorphism that conditions the CED phenotype of our two patients. In fact, patient 1 is homozygous for this RANKL change and is more severely affected than his heterozygous mother. Perhaps this exemplifies the influence of “other” genes on the variable CED phenotype, including an allele dosage effect of such an additional gene(s). It is not understood, however, how the novel TGFβ1 defect, and perhaps RANKL change, causes this unique variant of CED with widespread skeletal disease and histopathological changes of osteomalacia.
